# 

**Published:** 1870-01

**Authors:** 


### Commencement in the Buffalo Medical College.

The commencement exercises of the Buffalo Medical College will be held Tuesday evening, Feb. 22d, at the First Presbyterian church. The address to the graduating class will be delivered by Rev. Walter J. Clark, D. D., of Buffalo. It is believed the occasion will be one of interest to the profession and public, and all are invited to be present. It is hoped that curators of the College and members of the profession from the surrounding towns and villages who may be present at the College examinations, will make arrangements to remain and take part in the commencement exercises. J. F. MINER, M. D,

*Dean.*

———:o:———

### Erie County Medical Society Dinner.

As a matter for future reference, and as a fact in history, it should be distinctly stated, that Erie County Medical Society does not, and probably cannot, eat dinner; it is never hungry, and will not eat if it is.

Resolutions for a dinner, and committees to provide dinner, and various suggestions about a Society dinner, are often made, and made all very well, but no real dinner will be needed.

### Books and Pamphlets Received.

Disease and Injuries of the Eye: Their Medical and Surgical Treatment. By George Lawson, F. R. C. S., Surgeon to the Royal London Ophthalmic Society and Assistant Surgeon to the Middlesex Hospital. Lindsay & Blakiston, Philadelphia. For sale by T. Butler & Son.

Obstetric Aphorisms: For the use of Students commencing Midwifery Practise. By Joseph Griffiths Swayne, M D.,Physician Accoucheur to the Bristol General Hospital, etc. From the fourth revised English edition, with additions. By Edward R. Hutchins, M. D. Philadelphia, H. C. Lea. For sale by T. Butler & Son.

Transactions of the American Ophthalmological Society, Sixth annual meeting. Also of the American Otological Society, Second annual meeting.

Quarterly summary of the Transactions of the College of Physicians of Philadelphia.

Fourth Annual Report of the Metropolitan Board of Health of the State of New York.

Fourth Annual Report of the Trustees of the State Lunatic Asylum at Northampton, Massachusetts.

Report of the Committee on the Relations of Alcohol to Medicine. By John Bell, M. D., Chairman. From Transactions of American Medical Association.

Report of the Committee on the Result of Consanguineous Marriages. By Dr. Robert Newman of New York. Presented to the New York Medical Society.

Acupressure. By Joseph C. Hutchinson, M. D., Surgeon to Brooklyn City Hospital, etc. An Essay, to which was awarded a prize by the New York State Medical Society.

Plastic Operations. A paper read before the New York Academy of Medicine, February 18th, 1869. By Prof. Frank H. Hamilton.

The Physiological Action and Therapeutic Uses of "Acidum Phosphoricum Dilutum." By Judson P. Andrews, M. D., Assistant Physician in the New York State Lunatic Asylum.

Annual Announcement and Catalogue of the Albany Medical College. Thirteenth Session, 1869-70.

Annual Announcement of the Medical School of Maine, at Bowdoin College, for the course commencing February 14th, 1870.

Inaugural Address delivered at the Hahnemann Medical College of Philadelphia, at the opening of the session of 1869-70.

The Medical Temperance Journal. Published for the National Temperance League, by Wm. Tweedie, Strand, London.

Alcohol not Food: A reply to the statements of Dr. Thudicham before the Sub-Committee of the Society of Arts. By Henry Munroe, M. D., F. L. S , London.

The American Booksellers Guide. Published monthly, by the American News Company, 119 and 121, Nassau Street, New York City.

Albany Law Journal, Vol I, No. I.

Public Ledger Almanac for 1870. G. W. Childs, Philadelphia.

Vick's Illustrated Catalogue and Floral Guide for 1870. James Vick, Rochester, N. Y.

Prang's Chromo, a Journal of Popular Art.

The Woman's Journal. Vol. I, No. I. Boston.

